# c-Myb regulates transcriptional activation of miR-143/145 in vascular smooth muscle cells

**DOI:** 10.1371/journal.pone.0202778

**Published:** 2018-08-31

**Authors:** Mark Chandy, Masayoshi Ishida, Eric A. Shikatani, Omar El-Mounayri, Lawrence Changsu Park, Talat Afroze, Tao Wang, Philip A. Marsden, Mansoor Husain

**Affiliations:** 1 Ted Rogers Centre for Heart Research, University Health Network, Toronto, Canada; 2 Toronto General Hospital Research Institute, University Health Network, Toronto, Canada; 3 Department of Medicine, University of Toronto, Toronto, Canada; 4 Department of Physiology & Regenerative Medicine, Faculty of Medicine, Kindai University, Osaka-Sayama, Japan; 5 Li Ka Shing Knowledge Translation Institute, St. Michael’s Hospital, Toronto, Canada (LCP); 6 Departments of Physiology, University of Toronto, Toronto, Canada; Qatar University College of Health Sciences, QATAR

## Abstract

**Background:**

MicroRNAs (miR) are small non-coding RNAs that regulate diverse biological functions. The bicistronic gene miR-143/145 determines cell fate and phenotype of vascular smooth muscle cells (VSMC), in part, by destabilizing *Elk-1* mRNA. The transcription factor c-Myb also regulates differentiation and proliferation of VSMC, and here we test whether these effects may be mediated by miR-143/145.

**Methods & results:**

Flow cytometry of cardiovascular-directed d3.75 embryoid bodies (EBs) isolated smooth muscle progenitors with specific cell surface markers. In *c-myb* knockout *(c-myb* ^*-/-*^*)* EB, these progenitors manifest low levels of miR-143 (19%; p<0.05) and miR-145 (6%; p<0.01) expression as compared to wild-type (*wt*) EB. Primary VSMC isolated from transgenic mice with diminished expression (*c-myb*^*lx/lx*^) or reduced activity (*c-myb*^*h/h*^) of c-Myb also manifest low levels of miR-143 (*c-myb*^*lx/lx*^: 50%; *c-myb*^*h/h*^: 41%), and miR-145 (*c-myb*^*lx/lx*^: 49%; *c-myb*^*h/h*^: 56%), as compared to *wt* (P<0.05). Sequence alignment identified four putative c-Myb binding sites (MBS1-4) in the proximal promoter (PP) of the miR-143/145 gene. PP-reporter constructs revealed that point mutations in MBS1 and MBS4 abrogated c-Myb-dependent transcription from the miR-143/145 PP (P<0.01). Chromatin immunoprecipitation (ChIP) revealed preferential c-Myb binding at MBS4 (p<0.001). By conjugating *Elk-1* 3’-untranslated region (UTR) to a reporter and co-transducing *wt* VSMC with this plus a miR-143-antagomir, and co-transducing *c-myb*^*lx/lx*^ VSMC with this plus a miR-143-mimic, we demonstrate that c-Myb’s ability to repress *Elk-1* is mediated by miR-143.

**Conclusion:**

c-Myb regulates VSMC gene expression by transcriptional activation of miR-143/145.

## Introduction

MicroRNAs (miR) are conserved, small, non-coding RNAs that are 20–25 nt in length [1]. RNA polymerase II transcribes *pri*-miR that are processed by the nuclear RNase complex Drosha–DGCR8 into *pre*-miR, that are then translocated from the nucleus by Exportin5. In cytoplasm, Dicer processes pre-miR into mature miR that are subsequently loaded into RNA-induced silencing complex (RISC) [1]. miR modulate gene expression by destabilizing mRNAs or disrupting their translation, and thus serve as important regulators of events such as proliferation and differentiation [2,3]. In the vasculature, miR have been implicated in the differentiation of smooth muscle cell progenitors [4], and in the proliferation of adult vascular smooth muscle cells (VSMC) [5]. However, little is known about the transcriptional machinery that regulates miR in the production of VSMC from stem/progenitor cells or to what extent they contribute to the pathophysiology of cardiovascular disease. Understanding these regulatory processes may present them as therapeutic targets.

Embryonic stem cells (ESCs) can be differentiated into SMC lineages [6,7]. Although the precise roles of miR networks in their specification to SMC remain unclear, expression of miR-1, -10, -26a and -143/145 have been implicated [8–11]. In particular, the bicistronic gene miR-143/145 appears to regulate VSMC differentiation [12], with miR-143 and miR-145 destabilizing specific mRNAs important to the VSMC phenotype, such as platelet-derived growth factor receptor alpha (PDGFRα) and Kruppel-like factor 4 (KLF4) [13]. While miR-143 and miR-145 KO mice are viable, they have decreased levels of the VSMC markers SM α-actin, SM-MHC and smoothelin, and their phenotype includes reduced media thickness, arterial hypotension, and blunted responses to vasopressors [14]. Hence, miR-143 and miR-145 are important for VSMC differentiation, maintenance, and cell function with effects on integrated vascular pathophysiology.

The transcription factor c-Myb, first described as a key regulator of proliferation and differentiation of hematopoietic cell types [15,16], also plays an important role in the generation of VSMC from ESCs. ESCs give rise to vascular endothelial growth factor receptor type-2 (VEGFR2)-expressing progenitors, which can produce VSMC in a process involving c-Myb. Indeed, *vegfr2* KO embryoid bodies (EB) have decreased expression of Pim-1 kinase, a known modulator of DNA binding sites for c-Myb, resulting in limited formation of cells expressing SM-actin [17,18]. Furthermore, we found that *c-myb*^*-/-*^ ESC could be differentiated into cardiomyocytes but not contractile SMC in EB [19], and demonstrated that c-Myb not only activates VEGFR2 expression but also enables the subsequent capacity of VEGFR2^+^ progenitors to differentiate into VSMC [20]. Thus, both c-Myb and miR-143/145 serve important roles in ESC to VSMC differentiation that appear to have similar, or complementary, effects.

The roles of c-Myb and miR-143/145 in VSMC expansion holds true in the pathological state. Expansion of the VSMC compartment clearly underlies the pathogenesis of both vessel responses to injury and atherosclerosis [21,22]. The mechanisms by which this occurs include: proliferation of normally quiescent synthetic VSMC, the phenotypic modulation of differentiated, contractile VSMC to a synthetic cell type or a proliferative cell type [23], and the proliferation and subsequent differentiation of vessel-resident (or perhaps even circulating) progenitors of VSMC [14,24]. Supporting the latter, we recently showed that c-Myb regulates the proliferation and differentiation of adult *Sca1*^*+*^ adventitial VSMC progenitors, with the differentiation of this progenitor critically mediated by c-Myb-dependent transcriptional activation of myocardin [25]. Interestingly, miR-143 and miR-145 KO mice have impaired VSMC proliferation and medial thickening after carotid ligation [5], sharing a similar phenotype to that of mice with VSMC-specific expression of dominant-negative form of c-Myb (Myb-*Engrailed*) [26], and that of mice homozygous for a hypomorphic point mutation in the c-Myb transactivation domain (*c-myb*^*h/h*^) [25]. Given these observations, we hypothesized that the effect of c-Myb may be mediated, at least in part, by its ability to regulate expression of miR-143 and miR-145 in both embryonic and adult models of VSMC differentiation.

## Materials and methods

### ESC culture

Cytogenetic studies confirmed early (<10) passage *c-myb*^-/-^ ESCs of the CCE genetic background to harbor normal chromosomes (data not shown), and all studies used ESC from this stage. *wt* and *c-myb*^*-/-*^ CCE ESCs were maintained as previously described [20].

### Mouse cell lines and cell culture

Primary VSMC were isolated as described [27]. Primary VSMC were isolated from homozygous LoxP (Myb knockdown strain) mice (*c-myb*^*lx/lx*^) and their *wt* littermate controls. LoxP allele carrying mice [16] have a neomycin resistance (neo^R^) cassette inserted into the *c-myb* locus at intron-6 and also have LoxP sites inserted into introns-2 and -6 of the *c-myb* locus (*c-myb*^*lx*^). Insertion of the neo^R^ cassette into intron-6 of the c-*myb* gene results in an alternatively spliced event where mis-splicing of exon5 to the neoR cassette occurs with 90% probability and normal exon5-exon6 event splicing occurs with a 10% probability. Thus, the *c-myb*^*lx*^ allele produces only 5–10% of the *wild-type* level of full-length c-Myb protein, as validated by immunoblots of fetal liver protein extracts from E11 embryos probed with a c-Myb specific monoclonal antibody [16]. *c-myb*^*lx/lx*^ mice have a significantly smaller body size and a reduced lifespan; with most animals unable to survive past 4–6 months of age. A mouse carotid VSMC line derived from *c-myb*^*lx/lx*^ mice was immortalized as previously described [27]. Primary mouse carotid VSMC were cultured in DMEM with 10% fetal bovine serum, 50ng/mL rat recombinant PDGF-ßß (Sigma-Aldrich, Mississauga, ON), and 1% penicillin-streptomycin in humidified atmosphere with 5% CO2

### Site directed mutagenesis

To disrupt c-Myb binding site in the miR-143/145 promoter, MBS1 (ΔMBS1: aatTAACtgcatgct to aatTGGGtgcatgc), MBS2 (ΔMBS2: ggtCAACaggcattg to ggtCGGGaggcattg), MBS3 (ΔMBS3: atTAACtgcatgc to aatTGGGtgcatgc), and MBS4 (ΔMBS4: tgtCAACagcttgaa to tgtCGGGagcttgaa) were mutated using the Quickchange II Site Directed Mutagenesis Kit (#200523) and primers described in **S1 Table**. Each clone was confirmed with sequencing.

### Quantitative real-time RT-PCR

For RNA extraction and qRT-PCR of mRNA transcripts, total RNA from EBs and monolayers of mouse carotid VSMCs were isolated using the miRvana system (Lifesystems). miRNA was tested for purity (UV absorption ratio, A260/A280 > 1.8). DNase I (Fermentas) treatment was performed at room temperature for 30 min. RNA was reverse transcribed using qScript cDNA SuperMix (Quanta Biosciences). For microRNA qPCR, PerfeCTa SYBR Green SuperMix qPCR was employed with primers corresponding to the exact sequences of each individual microRNA (Integrated DNA Technologies, **S2 Table**) and the universal reverse primer supplied by the manufacturer (Quanta Biosciences). Samples were analyzed in triplicate. Final primer concentrations used were 0.2 μM with 60°C annealing temperature. Standard curves were generated for all primers and samples were run in triplicate. qPCR for mRNA and miRNA were performed on Roche LightCycler 480 System as per the manufacturer’s instructions, and normalized to expression of the housekeeping genes GAPDH or U6.

### Fluorescence-activated cell sorting (FACS)

FACS was performed as previously described [20]. In brief, EBs at indicated time were trypsinized and DNase treated. The cells were incubated with FITC-conjugated anti-mouse VEGFR2 (Flk1) (eBioscience) and PE-conjugated anti-mouse PDα (eBioscience) at 4°C for 30 min. The resultant cells were washed and sorted for each fraction (V-/P-, V+/P-, V+/P+, V-/P+) by flow cytometry performed with ARIA-RITT and LSR-SC (Becton-Dickinson). The FACS profiles were analyzed by FlowJo software (Ashland).

### Chromatin-immunopreciptiation (ChIP)

ChIP-IT Express Enzymatic Kit (Active Motif) was used as per the manufacture’s protocol. Mouse carotid VSMC and EB were fixed with 1% formaldehyde at room temperature for 10 min. Fixation was stopped with 125 mM glycine. Chromatin was isolated using a dounce homogenizer and centrifugation. After resuspending in digestion buffer, chromatin was enzymatically digested into 400-1500bp fragments, which were IPed overnight at 4°C with rabbit anti-c-Myb antibody (2μg, SC-517X, Santa Cruz Biotechnology), anti-H3 antibody or control rabbit serum (2μg, Santa Cruz Biotechnology). Chromatin-antibody complexes were pulled down with protein G-conjugated magnetic beads (Dynal, Invitrogen). IPed chromatin was eluted from the beads, reverse-crosslinked, treated with RNase-A and proteinase-K. ChIP was analyzed qualitatively by PCR and gel electrophoresis, and quantitatively with real time PCR with primers flanking putative c-Myb binding sites on a GeneAmp PCR System 9700 (Applied Biosystems) under the following conditions: 94°C 3 min, 94°C 30 sec, 57°C 30 sec, 72°C 30 sec for 40 cycles, and 72°C 30 sec. qPCR for ChIP were performed on Roche LightCycler 480 System. After determining the concentration of input chromatin by UV spectrometry, a standard curve was constructed for each set of primers. In this manner, qRT-PCR of each putative c-Myb binding site enabled us to quantify the absolute amount of c-Myb and/or H3 binding from the IPed fragment, which was then normalized to qRT-PCR results for ChIP performed with rabbit serum. The primer sequences used are provided in the **S3 Table**. qPCR data were analyzed by one-way ANOVA.

### Cell transfection and reporter gene assays

Immortalized VSMC, a mouse hemangioendothelioma endothelial cell line (EOMA), or human nasopharyngeal carcinoma (CNE2) cells were seeded at a density of 2.5 × 10^4^/well in a 6-well plate. At 18 h post seeding, the cells were washed once with phosphate-buffered saline, pH 7.4, and replaced with 2 ml of complete medium. Plasmids were transfected into EOMA using Jet Prime (Polyplus) and grown in high glucose Dulbecco's modified Eagle's medium (DMEM, Roche Applied Science) containing 5 u/ml penicillin, 50 μg/ml streptomycin, and 10% fetal bovine serum (FBS). The cells were incubated for 4 h with a total of 2 μg of plasmid DNA (1.5 μg of promoter-, 0.5 μg of various expression plasmids or empty vectors, and 0.05 μg of pRLTK-Renilla luciferase as an internal control). Media was subsequently removed and replaced with DMEM with 10% FBS and 5 u/ml penicillin. After 24 h, extracts (100 μl/well) were prepared for measurement of luciferase activity using Dual Luciferase Report (Promega, E1910). Cell lysates were plated onto a 96-well plate (Corning, 3917) and assayed on a PHERSTAR (BMG Labtech). Relative luminescence units over two ranges were recorded: Range-1: 2.5–11.5 secs or cycles 6–24 (Firefly luminescence) and Range-2: 14.5–23.5 secs or cycles 30–48 (*Renilla* luminescence). The level of firefly luciferase activity was normalized to control *Renilla* luciferase activity. Measurements were made from a minimum of three independent transfections. Results were reported as mean ± SEM, and all variables were analyzed by Students’ t-test, with significance defined as P ≤ 0.05.

### Immunoblots

At specified time intervals and after serum starvation and/or transient transfection, immortalized mouse carotid VSMC were harvested with PBS and pelleted by centrifugation for 10 min in a JS-4.2 rotor at 3000 rpm. The pellet was resuspended in hypotonic buffer (20 mM HEPES, pH 7.9, 25% glycerol, 1.5 mM MgCl2, 0.8 M KCl, 0.2 mM EDTA, 0.2 mM PMSF, 0.5 mM DTT) and incubated on ice for 30 min. Cell lysis was confirmed with microscopy and the cytoplasmic extract was removed by centrifugation at 18,000 RCF for 30 min at 4’C. The cell extracts were normalized using Lowry method and loaded on a 10% Tris Glycine gel and subjected to electrophoresis for 90 min at 125 V. Proteins were transferred to PVDF membranes and blocked in 5% milk with 1% BSA. The membrane was incubated with primary antibody (Elk-1 (1:1,000, Santacruz, sc-355), c-Myb (1:500, Santacruz, sc-517), sm22alph (1:1000, Santacruz, sc-53015) or GAPDH (1:10,000, Santacruz, sc-47724)) overnight at 4’C. The membranes were washed and incubated with donkey anti rabbit IgG (BioRad, #1706515) for 1 h at RT. The membranes were again washed and then incubated with Western ECL Lighting (Perkin-Elmer) for 1 min and detected on MiniBis Pro (Frogga Bio).

## Results

### c-Myb increases miR-143 and miR-145 expression in VSMC progenitor cells

**Fig 1:**
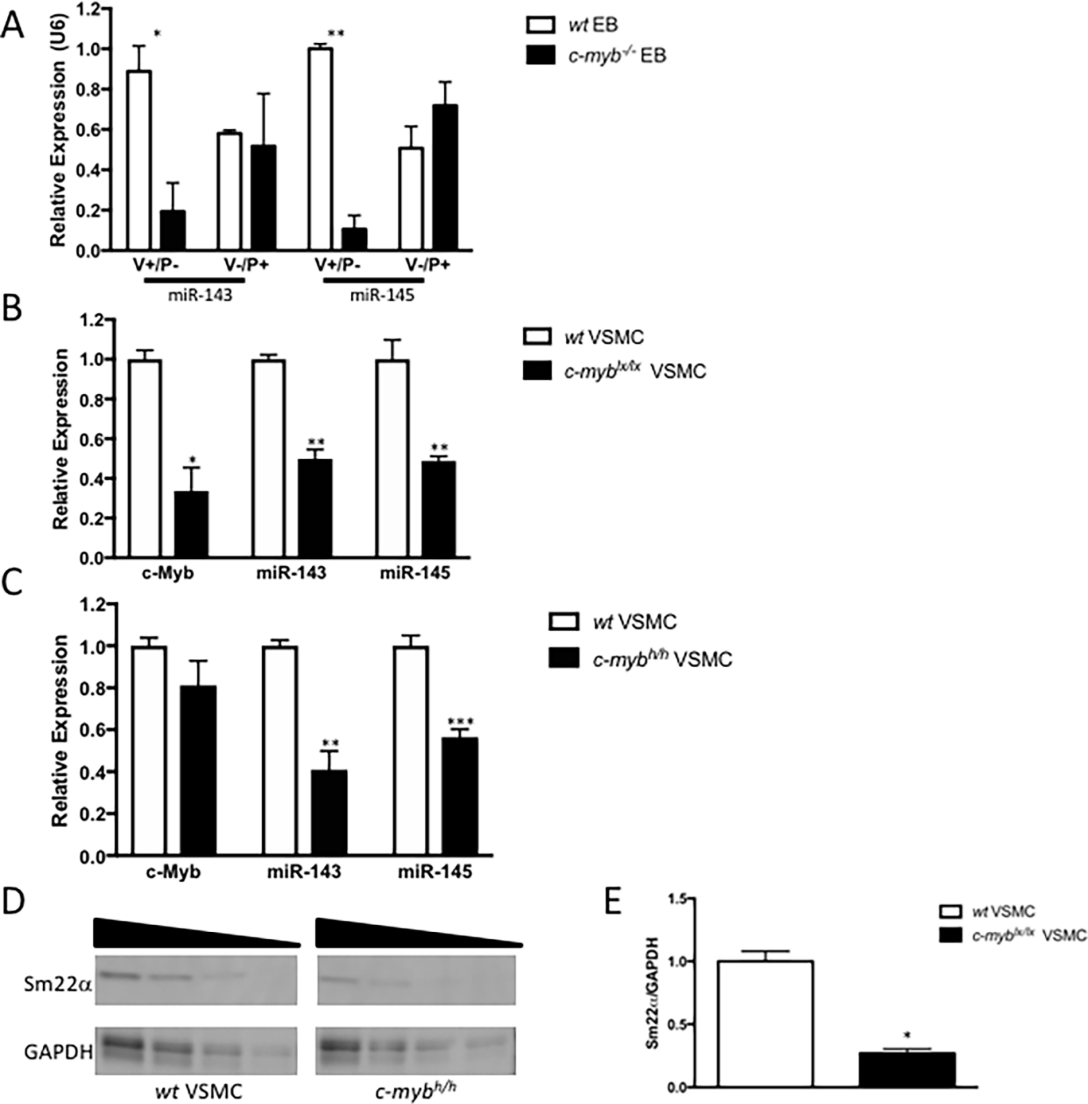
c-Myb-regulates expression of miR-143 and miR-145 in mouse embryonic stem cell-derived VEGFR2^+^ progenitors and adult mouse carotid vascular smooth muscle cells. **(A)** Flow cytometry of cardiovascular-directed d3.75 embryoid bodies (EBs), derived from *wild-type* (*wt*) or *c-myb*^*-/-*^ (knockout) mouse embryonic stem cells, was used to isolate progenitors with cell surface markers VEGFR2 (V) and PDGFRα (P). The known vascular smooth muscle cell (VSMC) progenitor V^+^/P^-^ derived from *c-myb*^*-/-*^ EB had lower expression levels of miR-143 (p<0.05*) and miR-145 (p<0.01**) than V^+^/P^-^ derived from *wt* EB. The non-SMC progenitor cells V^-^/P^+^ showed no such difference in miR-143 and miR-145 expression, compared to V^+^/P^-^. **(B)** Primary mouse carotid VSMCs isolated from *c-myb*^*lx/lx*^ mice have reduced expression levels of *c-myb* (p<0.05*), as well as miR-143 (p<0.01**) and miR-145 (p<0.001***) as compared to VSMCs isolated from *wt* mice. **(C)** Carotid VSMCs isolated from mice homozygous for a hypomorphic *c-myb* allele (*c-myb*^*h/h*^) have no differences in *c-myb* expression, but their known reductions in c-Myb activity were associated with reduced expression levels of miR-143 (p<0.01**) and miR-145 (p<0.001***) versus *wt*. qPCR analysis was performed with n = 3 biological samples and three technical repeats. **(D)** VSMC from *c-myb*^*lx/lx*^ mice show reduced expression levels of VSMC marker, Sm22α versus *wt*. **(E)** Densitometry reveals *c-myb*^*lx/lx*^ mice have significantly reduced expression of Sm22a relative to GAPDH compared to *wt* (p<0.05*, n = 4).

To assess the expression of miR-143 and -145 in ESC-derived VSMC progenitor cells, embryoid bodies (EB) from *wt* and *c-myb*^*-/-*^ mouse ESC were differentiated into VSMC progenitor cells under serum-free cardiovascular-directed conditions, and subsequently analyzed by flow cytometry for cell surface markers VEGFR2 (V) and PDGFRα (P) [20] to identify VSMC and non-VSMC progenitor populations. Given that c-Myb expression had been shown to peak at d3.75 of this protocol [20], miR-143 and miR-145 expression levels were also measured at this time point. *c-myb*^*-/-*^ EB-derived V+/P- progenitors, which generate functional smooth muscle cells [20], manifest markedly reduced expression levels of miR-143 (19%; p = 0.03) and miR-145 (6%; p = 0.003) when normalized to *wt* EB (i.e. 81% and 94% reduction in miR-143 and miR-145, respectively). By contrast, V-/P+ progenitors, which do not yield functional smooth muscle cells [20], had no change in miR-143/145 expression in *c-myb*^*-/-*^ EB *vs*. *wt* (n = 3) (**Fig 1A**).

We next tested the relationship between c-Myb expression or activity and miR-143 and miR-145 expression levels in primary VSMC isolated from the adult mouse carotid artery. Primary carotid VSMCs isolated from homozygous *c-myb*^*lx/lx*^ mice expressed lower levels of *c-myb* than VSMC isolated from *wt* littermates (**Fig 1B**). These carotid VSMC from *c-myb*^*lx/lx*^ mice also showed 50% lower miR-143 (p<0.01) and 49% less miR-145 (P<0.001) expression than primary carotid VSMC isolated from *wt* littermates. We next examined the relationship between *c-myb* and miR-143/145 in mice homozygous for a c-Myb hypomorphic allele (*c-myb*^*h/h*^), which carries a single point mutation in the activation domain of c-Myb but does not affect c-Myb expression levels. Compared to *wt*, aortas of c-*myb*^*h/h*^ mice show 41% less miR-143 (p<0.01) expression and 56% less miR-145 (p<0.01) expression than *wt* controls (**Fig 1C**).

To further examine the relationship between c-Myb, miR-143 and miR-145, and VSMC, we next assessed expression levels of the VSMC marker Sm22α in *c-myb*^*lx/lx*^ mice. Sm22α protein expression was 71% lower in primary carotid VSMC from *c-myb*^*lx/lx*^ *vs*. *wt* mice **(Fig 1D and 1E and S1 Fig**). Taken together, these data consistently suggest that c-Myb expression and activity correlate with expression levels of miR-143 and miR-145 in ESC-derived VSMC progenitors and adult VSMC, as well as with expression levels of the VSMC marker Sm22α.

### Specific DNA-binding sites on the miR-143/145 promoter mediate c-Myb-dependent transcriptional activation

**Fig 2:**
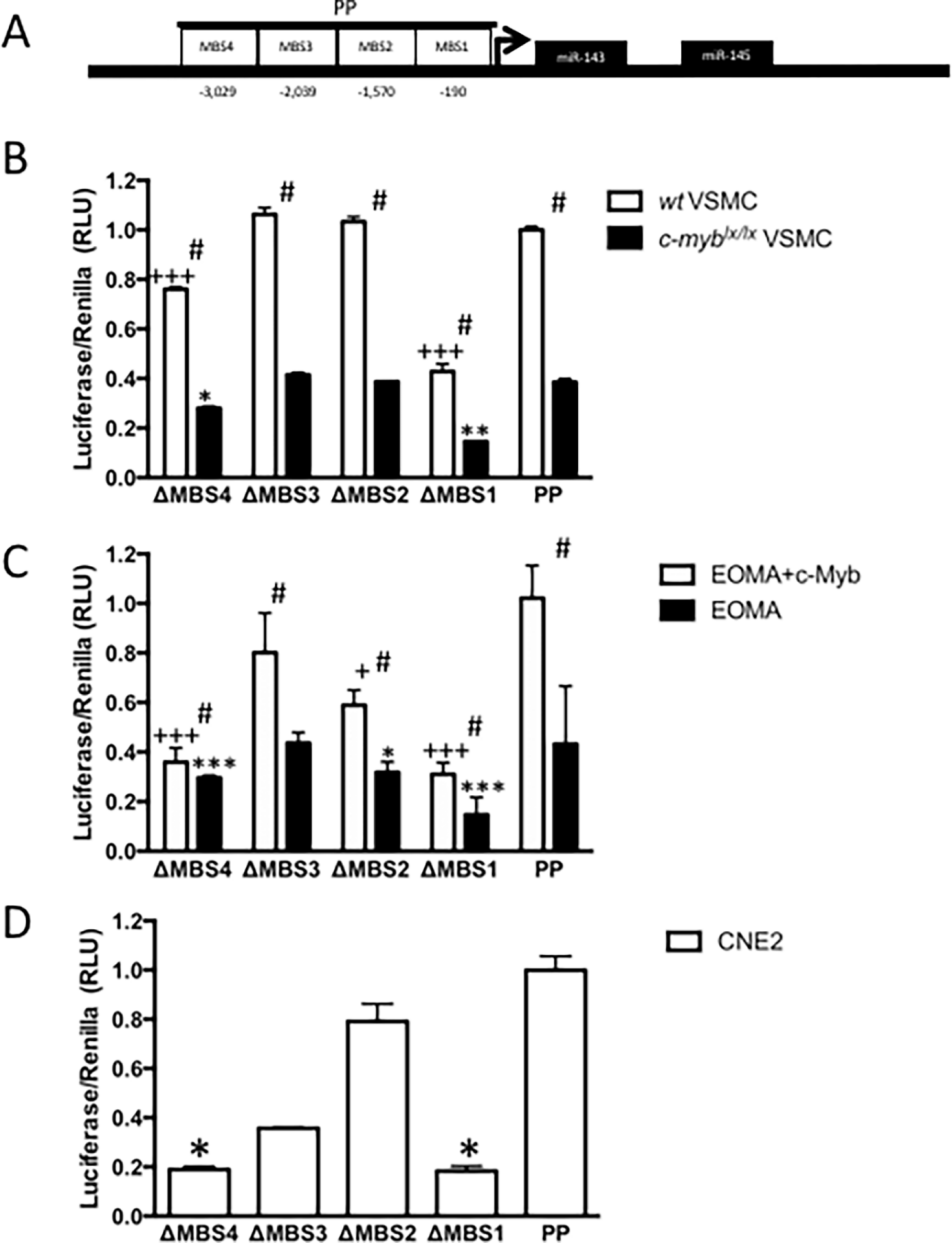
miR-143/145 promoter activity requires c-Myb binding sites (MBS). Promoter activity was determined by co-transfection with promoter-luciferase constructs and renilla control plasmid. Site-directed mutagenesis revealed MBS to be important for reporter gene activation when compared to intact proximal promoter. **(A)** Proximal promoter (PP) with c-Myb binding site (MBS) positions shown relative to predicted transcriptional start. **(B)** Promoter luciferase constructs were transfected in *c-myb*^*lx/lx*^ VSMC or *wt* controls. Compared to proximal promoter, MBS1 (p<0.001^+++^) and MBS4 (p<0.001^+++^) were required for c-Myb-dependent transcription in *c-myb*^*lx/lx*^ VSMC (n = 3), both MSB1 (p<0.01**) and MBS4 (p<0.05*) were also required for transcriptional activity in *wt* VSMC (n = 3). Importantly, activation in *wt* was always greater than in *c-myb*^*lx/lx*^ VSMC (p<0.05). **(C)** In EOMA, an endothelial cell line that does not express c-Myb, MBS1 (p<0.001***^/+++^), MBS2 (p<0.05*^/+^), and MBS4 (p<0.001***^/+++^) were relevant to transcriptional activity when compared to promoter, regardless of c-Myb expression (n = 12). Co-transfection with exogenous c-Myb is however, associated with increased transcriptional activity (p<0.05, n = 3). **(D)** The human nasopharyngeal carcinoma cell line (CNE2) is known to express c-Myb. Intact MBS1 (p<0.05*) and MBS4 (p<0.05*, n = 3) were also necessary for miR-143/145 promoter activity in this cell line.

An *in silico* analysis with MatInspector identified four conserved putative c-Myb-binding sites (MBS) in the proximal promoter (PP) of the miR-143/145 gene (**Fig 2A**). These MBS were located within a 4kb segment (**S2 and S3 Figs**). This PP includes the CArG box that putatively binds myocardin and an adjacent SMAD-binding element previously shown to regulate miR-143 and miR-145 expression [28]. The functional importance of these putative MBS was studied using site-directed mutagenesis of a promoter-luciferase reporter construct transfected into (a) carotid VSMC from *wt* mice, (b) EOMA, an endothelial cell line with no detectable *c-myb* expression in which c-Myb was also over-expressed, and (c) CNE2, which is known to express high levels of c-Myb [29].

In VSMC, the *wt* PP-reporter construct showed endogenous transcriptional activity (**Fig 2A**). Mutation of MBS1 and MBS4 in the PP caused a significant decrease in transcriptional activity in immortalized *wt* and *c-myb*^*lx/lx*^ VSMC (p<0.001 and p<0.001, respectively) (**Fig 2B**). Importantly, *wt* VSMC manifest higher levels of PP-reporter activity as compared to *c-myb*^*lx/lx*^ VSMC (p<0.05). Next, EOMA cells cotransfected with c-Myb show significantly greater miR-143/145 promoter activity as compared to EOMA cells without c-Myb expression (p<0.05), and point mutations in MBS1 (p<0.001), MBS2 (p<0.05) and MBS4 (p<0.001) were found to be functionally important in this cell line (**Fig 2C**).

Using CNE2 cells, Wang *et al*. had demonstrated that c-Myb bound the miR-143 and miR-145 promoter, but purportedly functioned as a repressor [29]. In our analysis, c-Myb binding sites MBS1, MBS2 and MBS3 were identical to those identified by Wang *et al*. (**S2 Fig**), while MBS4 was not identified in their analysis. However, in our hands, c-Myb *activates* the miR-143/145 promoter in CNE2 cells, with point mutations in MBS1 (p<0.05) and MBS4 (p<0.05) significantly reducing this activity (**Fig 2D**). Finally, further supporting c-Myb-dependent transcriptional activation of the miR-143/145 gene, qRT-PCR analysis of VSMC isolated from *c-myb*^*lx/lx*^ mice revealed reduced *pre*-miR-143 and *pre*-miR-145 levels as compared to *wt* controls (**S4 Fig**). Thus, we demonstrate, with multiple cell lines from different species, that c-Myb is functioning as a transcriptional activator of the miR-143/145 gene.

### c-Myb binds to the promoter of the miR-143/145 gene

**Fig 3:**
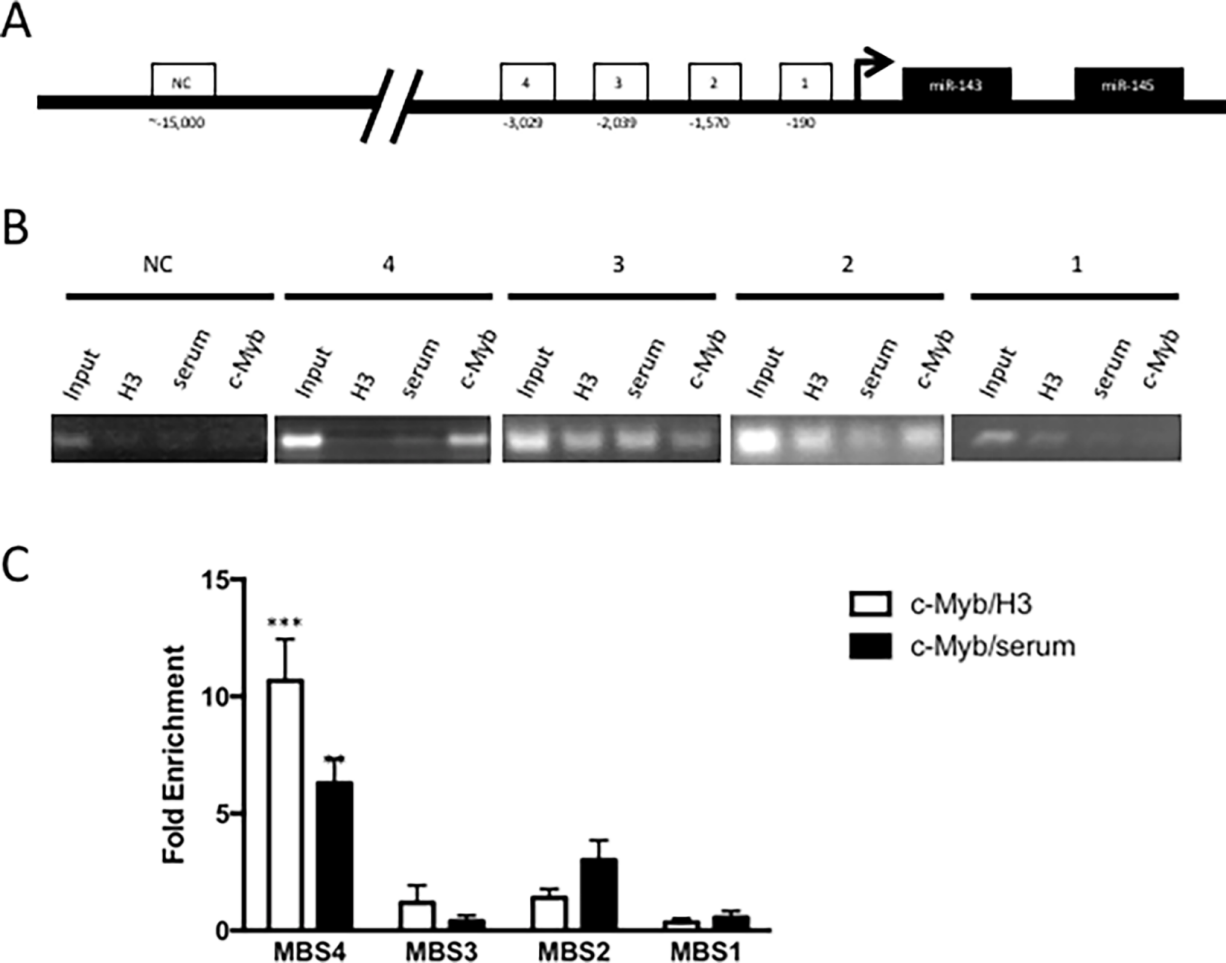
Chromatin immunoprecipitation (ChIP) of c-Myb on the miR-143/145 promoter of immortalized mouse carotid artery vascular smooth muscle cells (VSMC). Cells were fixed, lysed and chromatin was fragmented into oligonucleosomes (200-1500bp) then immunoprecipitated overnight at 4°C with rabbit anti-c-Myb antibody (2mg, SC-517X, Santa Cruz), anti-histone H3 (2mg, Santa Cruz) or control rabbit serum (2mg, Santa Cruz). **(A)** The miR-143 and miR-145 promoter contains four c-Myb binding sites (MBS). **(B)** ChIP and PCR revealed specificity of c-Myb binding to MBS in carotid VSMC. As a negative control, ChIP was performed on a promoter situated ~15kb upstream of miR-143/145 with no known or predicted MBS. **(C)** qPCR reveals differential binding of c-Myb on predicted MBS of the miR-143/145 promoter. c-Myb preferentially binds MBS2 and MBS4 in carotid VSMC (p<0.01, one-way ANOVA). When normalized to histone H3 density, c-Myb preferentially binds MBS4 (p<0.001***, one-way ANOVA). ChIP was performed in n = 3 biological samples. qPCR analysis had three technical repeats for each biological sample.

Chromatin immunoprecipitation (ChIP) analysis was used to detect histone H3 and c-Myb binding at the miR-143/145 promoter in carotid VSMC (**Fig 3A**). c-Myb was easily IPed on the miR-143 and miR-145 promoter. By PCR and agarose gel electrophoresis, c-Myb appears to bind MBS4 and MBS2 (**Fig 3B**) (p<0.01). By contrast, histone H3 occupancy was not statistically distinct at any MBS (data not shown). When c-Myb binding is normalized to histone H3 binding, c-Myb can be shown to preferentially bind only MBS4 in *wt* VSMC (one-way ANOVA, p<0.001) (**Fig 3C**). As a negative control (NC), a promoter without any putative c-Myb binding sites was subjected to ChIP with the same c-Myb antibody (**Fig 3A**). In this experiment, no c-Myb binding can be detected on this NC promoter (**Fig 3B**). These data reveal that c-Myb targets miR143/145 by preferential binding of MBS4.

### c-Myb regulates Elk-1 expression via miR-143

**Fig 4:**
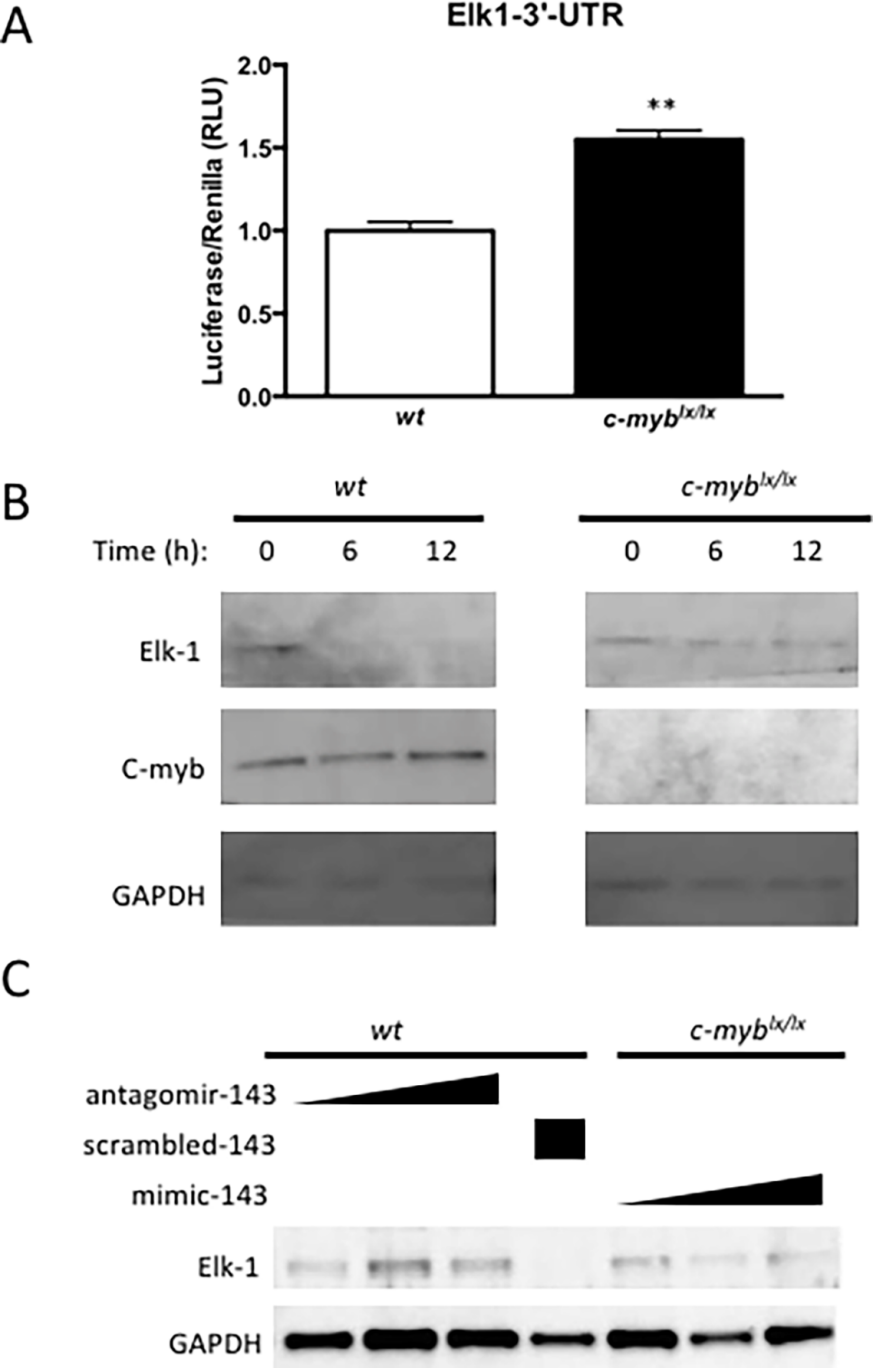
c-Myb represses Elk-1 expression via miR-143/145. **(A)** Elk1 has previously been validated as a miR-143/145-repressed gene. Elk1-3’UTR-Luciferase construct was transfected into *wt* and *c-myb*^*lx/lx*^ carotid artery VSMC. Luciferase activity was assayed on PHERASTAR and found to be augmented in *c-myb*^*lx/lx*^ VSMC, which have known reductions in c-Myb expression (p<0.01**). **(B)** Time course analysis of c-Myb and Elk-1 expression. After serum starvation and cell cycle arrest, c-Myb levels are diminished. Following addition of 10% fetal bovine serum, c-Myb expression peaks at 12 h. As c-Myb expression level rise, Elk-1 expression levels decrease and are undetectable at 12 h. Note that c-Myb expression is not detectable in *c-myb*^*lx/lx*^ VSMC released from serum starvation, and Elk-1 expression remains stable. **(C)** miR-143 mediates c-Myb repression of Elk1. The miR-143 antagomir augments Elk-1 protein expression in *wt* cells that express c-Myb, while the mimic abrogates protein expression in *c-myb*^*lx/lx*^ VSMC.

The activity and expression of *Elk-1*, a known target of miR-143 [12] that is involved in the regulation of VSMC proliferation [30], were assessed to investigate the c-Myb-mediated regulation by miR-143 on downstream VSMC regulators. By conjugating the 3’ untranslated region of *Elk-1* to a luciferase reporter (3’UTR-*Elk-1*-luc), the stability of this mRNA, as assessed by luciferase activity, can be used to gauge the consequences of transfection with a miR-143 mimic and conversely with an antagomir to miR-143. In this manner, the effect of c-Myb activity on miR-143-driven 3’UTR-*Elk-1*-luciferase activity was assayed in *c-myb*^*lx/lx*^ and *wt* VSMC. Reflecting *reduced* miR-143 destabilization of 3’UTR-Elk-1-luc, luciferase activity was greater in *c-myb*^*lx/lx*^ than *wt* VSMC (p<0.01) (**Fig 4A**).

Upon serum starvation-induced cell cycle arrest, immunoblots reveal reduced c-Myb expression in VSMC. These levels of c-Myb expression rapidly increase upon the addition of fetal bovine serum (FBS) and peak at 12 h (**Fig 4B**). Coincident with this, c-Myb-driven miR-143-mediated Elk-1 protein expression is dramatically reduced in serum-stimulated *wt* VSMC. By contrast, *c-myb*^*lx/lx*^ VSMC with undetectable levels of c-Myb do not suppress Elk-1 expression following serum stimulation (**Fig 4B**). These presumed miR-143-mediated effects on Elk-1 expression were then tested with miR-143 mimic and antagomir experiments in *c-myb*^*lx/lx*^ and *wt* VSMC. miR-143 antagomir abrogates the effect of c-Myb of Elk-1- expression in *wt* VSMC. By contrast, a scrambled oligonucleotide (negative control for the antagomir) had no such effect. Conversely, *c-myb*^*lx/lx*^ VSMC transfected with a miR-143 mimic show reduced Elk-1 expression, even in the absence of normal c-Myb levels (**Fig 4C**). These data demonstrate that c-Myb represses Elk-1 via miR-143.

## Discussion

We have identified c-Myb as a transcription factor that regulates miR-143 and miR-145 expression levels in both ESC and adult VSMC. c-Myb is required for miR-143 and miR-145 expression in ESC-derived VEGFR2^+^ progenitor cell populations. In adult mouse carotid VSMC, *c-myb*^*lx/lx*^ VSMC with diminished levels of c-Myb expression have reduced levels of miR-143 and miR-145. Of the multiple MBS identified by *in silico* analysis of the PP of the miR-143/145 gene, we show that while MBS1 and MBS4 are important for transcriptional activation, only MBS4 is found to bind c-Myb in *wt* VSMC. Our study also demonstrates that c-Myb-dependent regulation of miR-143 has functional consequences in VSMC, with c-Myb-dependent regulation of Elk-1 gene expression and subsequent translation being directly mediated by a miR-143-responsive destabilizing motif in its 3’-UTR.

### c-Myb activates transcription on the miR-143 and miR-145 promoter

Although a recent study has suggested that c-Myb can bind and regulate the miR-143/145 promoter in a nasopharyngeal carcinoma cell line (CNE2), they found c-Myb to be acting as a transcriptional repressor [29]. While c-Myb is capable of acting as both a transcriptional activator [31] and repressor [27], in our hands the miR-143/145 gene is *activated* by c-Myb in (a) in EB-derived VSMC progenitors, (b) adult VSMC, (c) the EOMA cell line transduced to express c-Myb, and (d) the CNE2 cell line. In EOMA, a cell line with no c-Myb expression, MBS mutations have decreased transactivation, which may be caused by other transcription factors that overlap with the c-Myb binding site. With respect to the CNE2 cell line, our data contradict those of Wang *et*. *al*. [29].

Wang *et*. *al*. describe c-Myb activating miR-143/145 in their abstract, but the results and subsequent discussion suggests c-Myb is a repressor. The data supporting the role of c-Myb as a repressor is based on correlations among nasopharyngeal cancer lines with high c-Myb and low miR-143/145 levels as determined by qRT-PCR. Anti-c-Myb siRNA caused a decrease in c-Myb mRNA and an increase in miR-143 expression, although anti-c-Myb siRNA may de-repress c-Myb autoregulatory repression mediated by dimerization [32]. Although Wang *et al*. also showed that c-Myb binds to the miR-143/145 promoter, their study did not capture MBS4. By contrast, our data clearly show MBS4 to be the most important binding site for c-Myb, with point mutations in MBS4 abrogating c-Myb-dependent transcriptional activation of the luciferase reporter. Our data also demonstrated that MBS1 and MBS2 were important for transcriptional activity, but MBS1 did not bind c-Myb. We speculate that MBS1 is in close proximity to the transcriptional start site and may not be associated with the active promoter. Curiously, Wang *et al*. described luciferase reporter experiments but did not provide data supporting this claim in either their manuscript or supplemental data online.

### c-Myb-directed VSMC progenitor differentiation via miR-143/145

Previous studies from our lab show high c-Myb expression in VEGFR2^+^ progenitor cells and that c-Myb is crucial for their differentiation from ESCs, with *c-myb*^*-/-*^ EB yielding greatly reduced numbers of VEGFR2^+^ progenitors [20]. In the current study, we go on to show that c-Myb mediates the effect of these miR, and that VEGFR2^+^ progenitor cells derived from *c-myb*^*-/-*^ EB have reduced expression of miR-143/145. Complementing our present work, other studies have implicated miR-143/145 in the formation of VEGFR2^+^ progenitors, suggesting that miR-143/145 may be sufficient to drive differentiation of ESC into VSMC progenitors [5,12,33].

### c-Myb regulates VSMC proliferation through miR-143 and miR-145

Several studies have demonstrated a role for increased c-Myb expression in remodeling responses following vascular injury [26,34]; however, there is discordance over the role of miR-143 and miR-145 in similar models. In a carotid ligation mouse model, Xin *et al*. demonstrated that miR-143 and miR-145 knockout mice have reduced neointima formation after vascular injury [5]. Conversely, Cheng *et al*. showed in a balloon injury rat model that a miR-145 mimic has the same effect [35]. In Apolipoprotein E-deficient mice, atherosclerotic lesions are associated with low miR-145 expression and plaque stability is improved with mimic therapy [36]. As such, we posit that the effects of miR-143 and miR-145 may be model- and context-specific and may not precisely reflect similar processes mediated by c-Myb.

The E26 transformation specific (ETS) family of transcription factors function in cell differentiation, proliferation, and angiogenesis [37]. ETS transcription factor Elk-1 antagonizes the effect of miR-145 and myocardin. Elk-1 blocks myocardin and promotes VSMC proliferation, while miR-145 and myocardin promote VSMC differentiation [37]. In the current study, we demonstrate that c-Myb-driven repression of Elk-1 is mediated by miR-143. Elk-1 is a known target of miR-143, functioning to prevent VSMC proliferation and to maintain quiescence [12].

In pathological conditions such as atherosclerosis and vessel injury, which are associated with a shift from quiescent to proliferative VSMC phenotypes, c-Myb expression levels increase [26,38], while miR-143 and miR-145 expression levels are reduced. These observations suggest directionally distinct regulation of c-Myb and miR-143/145 in *diseased* models, as opposed to our current demonstration of directionally parallel changes in c-Myb expression and activity and miR-143/145 expression. In this context, Elk-1 would be *de-repressed*, which would inhibit myocardin and promote the synthetic VSMC phenotype [12,35] that characterizes these conditions. Other results by Xin *et al*. and Cheng *et al*. add to the notion that c-Myb-dependent regulation of miR-143/145 is context-dependent [5,35]. One explanation stems from the potential role played by c-Myb in post-transcriptional regulation of miR by Dicer, which may itself be perturbed in atherosclerosis [39]

## Supporting information

S1 Fig Raw image of immunoblot from Fig 1 of Sm22a and GAPDH with titration of *wild-type* (*wt*) and *c-myb*^*lx/lx*^ VSMC protein extract. (TIFF)

S2 Fig miR-143/-145 promoter has multiple c-Myb binding sites. The putatively common miR-143 and -145 promoter was analyzed using JASPAR in VSMC derived from mouse, rat, and human. This analysis revealed four putative c-Myb binding sites.
(TIFF)

S3 Fig Histone K27 acetylation profile map of miR-143/145 promoter. https://genome.ucsc.edu/cgi-bin/hgTracks?db=mm10&lastVirtModeType=default&lastVirtModeExtraState=&virtModeType=default&virtMode=0&nonVirtPosition=&position=chr18%3A61645878%2D61665538&hgsid=596032207_KLqkDOTr888OArEskkTNXv4UJrO1.
(TIFF)

S4 Fig Pre-miR-143 and pre-miR-145 expression is decreased in *c-myb*^*lx/lx*^ compared wild (*wt*) cells (p<0.05*). (TIFF)

S1 Table Primer sequences for site directed mutagenesis for c-Myb binding sites in the miR-143/145 promoter. (TIFF)

S2 Table Primer sequences for quantitative real time PCR of microRNA-143/145. (TIFF)

S3 Table Primer sequences for ChIP analysis of the miR-143/145 promoter. (TIFF)
