# Supplementary figures and images for: c-Myb regulates transcriptional activation of miR-143/145 in vascular smooth muscle cells
Source: PLoS One. 2018 Aug 31;13(8):e0202778. doi: 10.1371/journal.pone.0202778 (PMC6118359; doi:10.1371/journal.pone.0202778)

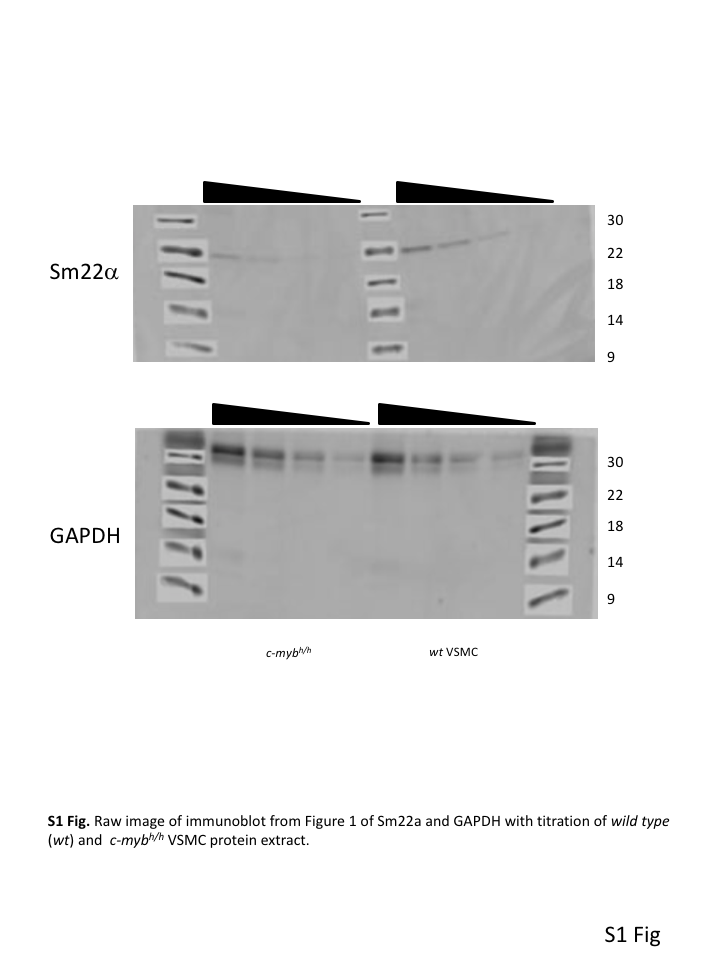

Supplement: S1 Fig — (TIFF) [file pone.0202778.s001.tiff]

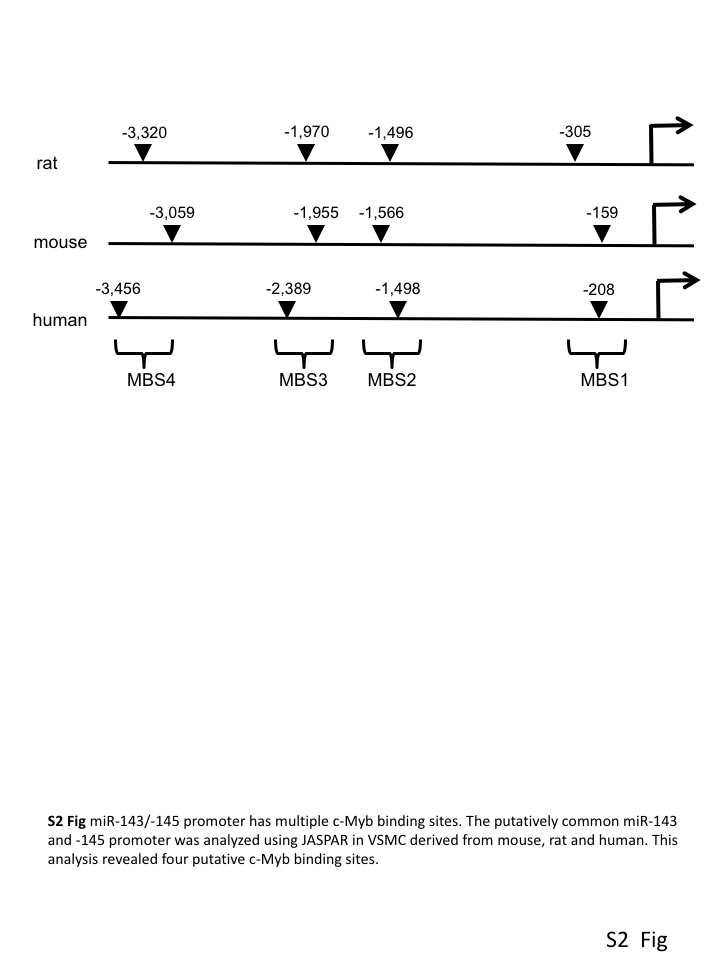

Supplement: S2 Fig — The putatively common miR-143 and -145 promoter was analyzed using JASPAR in VSMC derived from mouse, rat, and human. This analysis revealed four putative c-Myb binding sites. (TIFF) [file pone.0202778.s002.tiff]

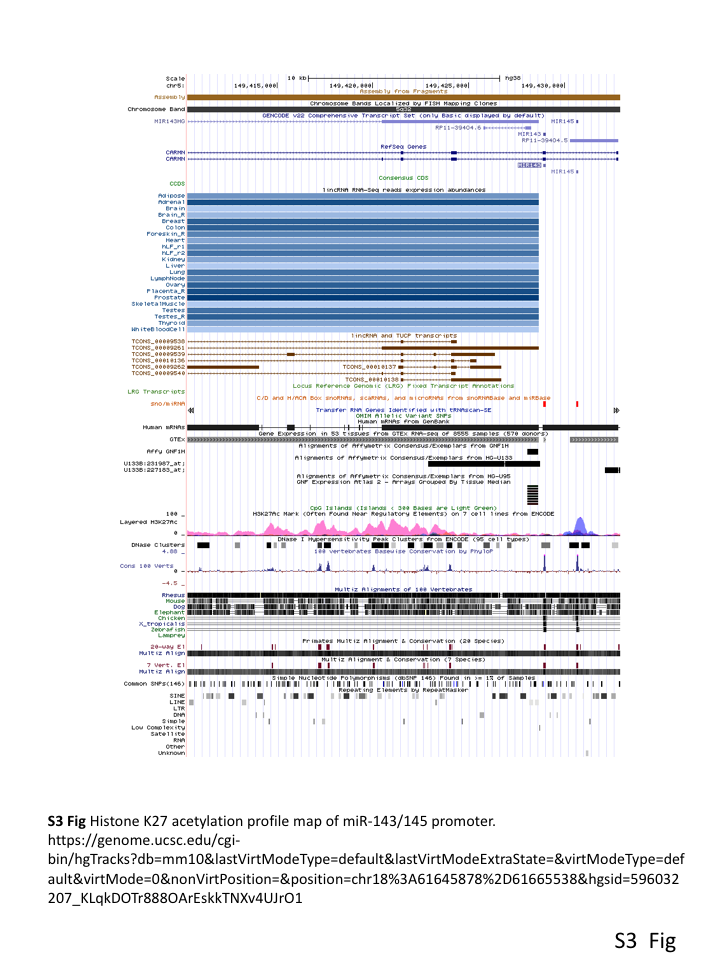

Supplement: S3 Fig — https://genome.ucsc.edu/cgi-bin/hgTracks?db=mm10&lastVirtModeType=default&lastVirtModeExtraState=&virtModeType=default&virtMode=0&nonVirtPosition=&position=chr18%3A61645878%2D61665538&hgsid=596032207_KLqkDOTr888OArEskkTNXv4UJrO1. (TIFF) [file pone.0202778.s003.tiff]

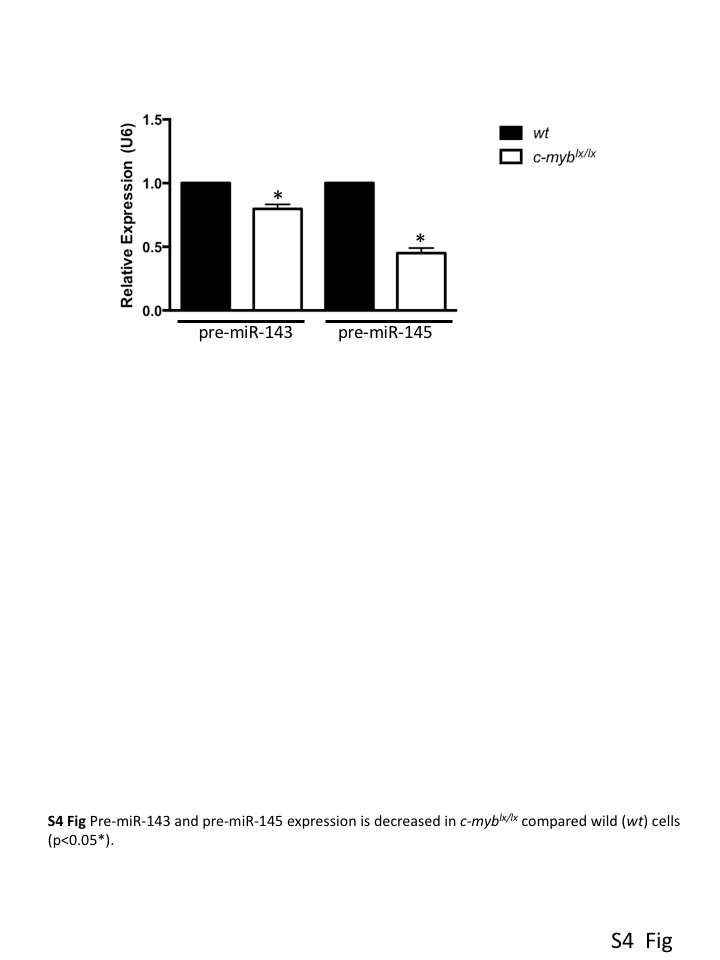

Supplement: S4 Fig — (TIFF) [file pone.0202778.s004.tiff]

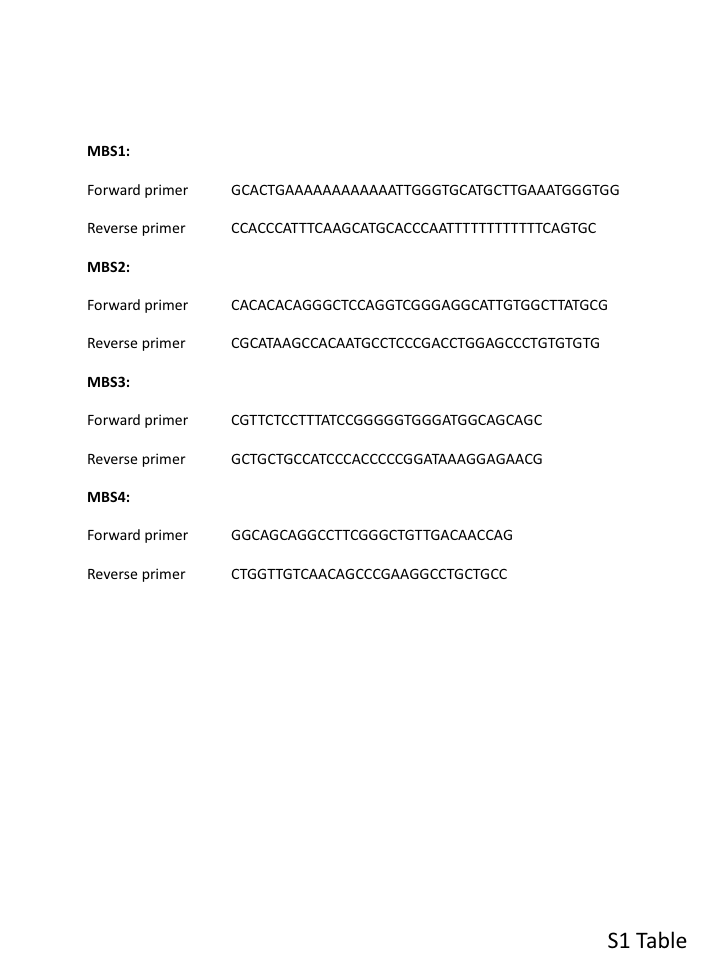

Supplement: S1 Table — (TIFF) [file pone.0202778.s005.tiff]

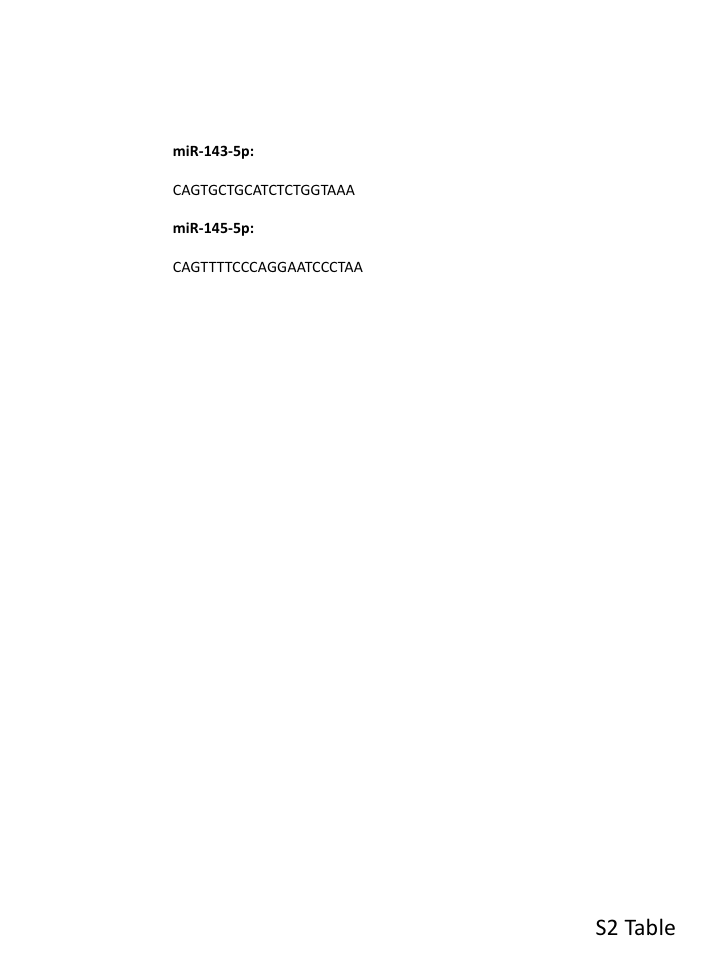

Supplement: S2 Table — (TIFF) [file pone.0202778.s006.tiff]

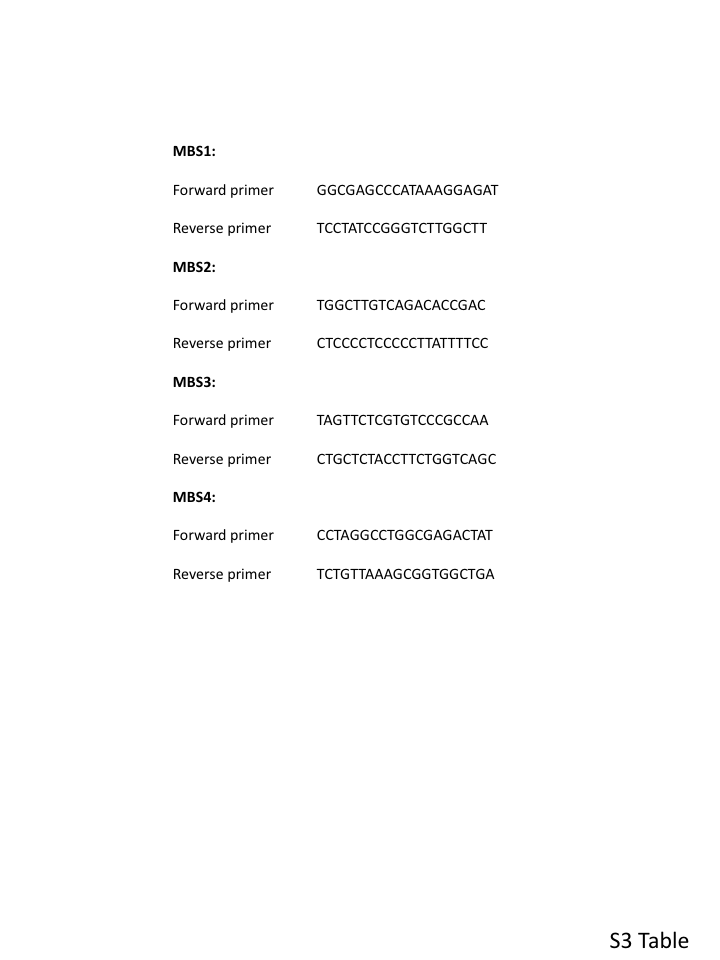

Supplement: S3 Table — (TIFF) [file pone.0202778.s007.tiff]
